# Betaine: A Promising Natural Product for Neurological and Psychiatric Diseases

**DOI:** 10.2174/011570159X375540250718094903

**Published:** 2025-08-08

**Authors:** Ying Zhang, Zhaojuan Ke, Jie Luo, Qibin Chen, Xin Jiang, Jialin Xiong, Linya Deng

**Affiliations:** 1 Department of Anesthesiology, The First Affiliated Hospital of Chongqing Medical University, Chongqing, China

**Keywords:** Betaine, neuropsychiatric disease, mental disorder, osmolyte, methyl donor, gamma-aminobutyric acid

## Abstract

Neurological and psychiatric diseases pose a considerable global burden. Exploring additional potential prevention strategies and therapies is ongoing. As a prevalent natural product and nutraceutical from food, betaine’s pharmaceutical applications suggest benefits for both health and disease in multiple organs. Recently, its efficacy on neurological and psychiatric health has been proposed and has drawn considerable attention. This review aims to provide an updated, critical, and comprehensive profile of the promising medicinal roles of betaine in these diseases. In addition to its well-known osmotic protection, due to methyl donation, it regulates metabolism, alleviates oxidative stress, and reduces inflammation. To manifest neurological and psychiatric health benefits, betaine acts by affecting gamma-aminobutyric acid associated with its transporters, related neurotransmitters, downstream and neurological pathways, and other specific mechanisms in the nervous system. Betaine demonstrates therapeutic potential against various neurological and psychiatric diseases, such as epilepsy, neurocognitive disorders (including Alzheimer's disease), Parkinson's disease, stroke, multiple sclerosis, traumatic brain injury, depression, anxiety, schizophrenia, autism spectrum disorder, sleep disorders, fetal alcohol syndrome, syringomyelia, neonatal brain injury, neuropathic pain, and motor dysfunction. Despite the promising role of betaine in the treatment, diagnosis, and prevention of neuropsychiatric disorders, much of the present evidence appears to be fragmentary. Further studies elucidating the underlying mechanisms and direct clinical applications are required to obtain a deeper understanding of betaine and its underutilized potential. Overall, this review highlights the potential of betaine as a promising agent with benefits for neurological and psychiatric diseases, aiming to offer clues to advance this field.

## 1 INTRODUCTION

**Fig. (1):**
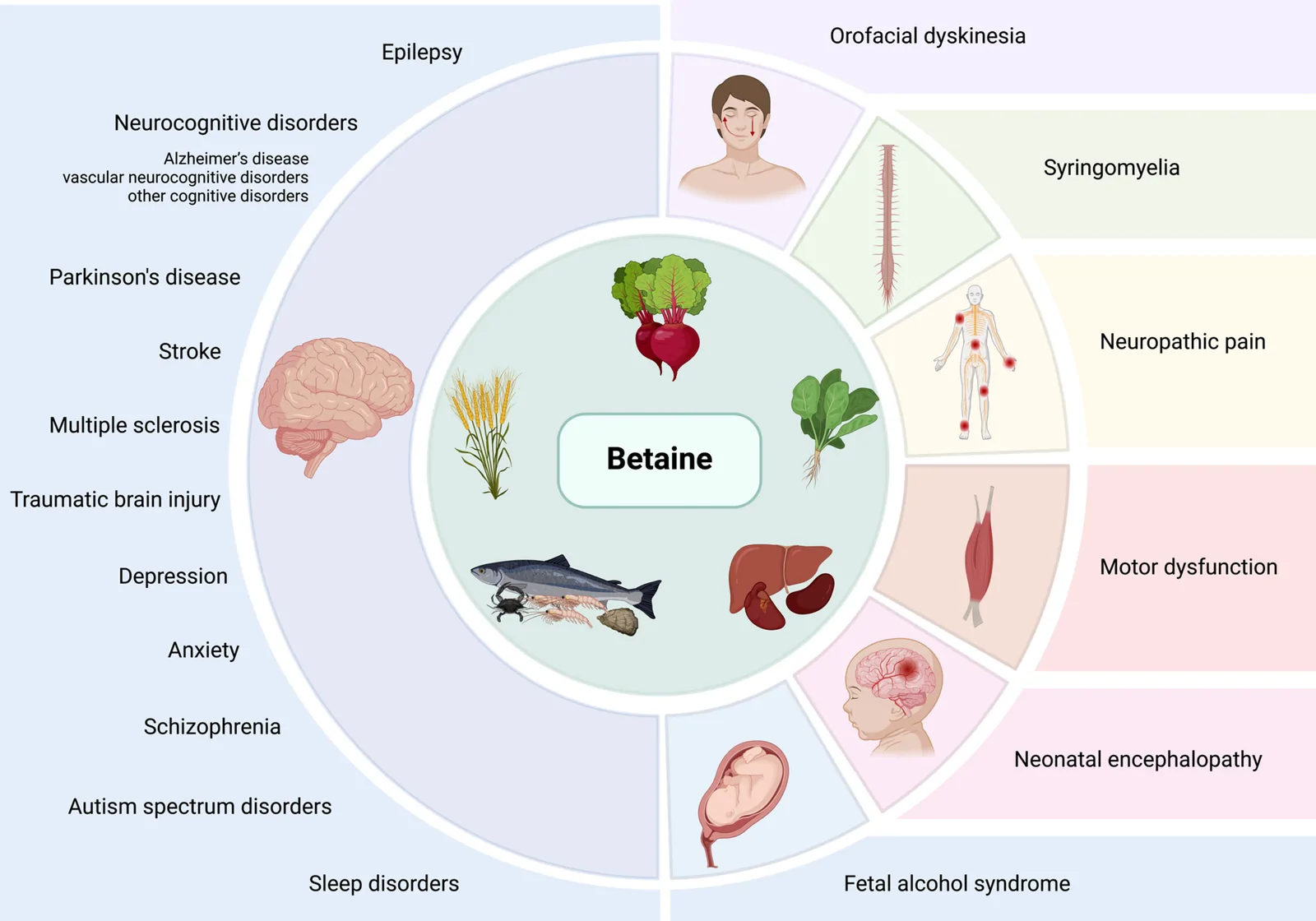
Betaine and the neurological and psychiatric related diseases that can be benefitted from it.

A natural product and conventional nutraceutical from food, betaine (trimethylglycine, (CH_3_)_3_N^+^CH_2_COO^-^), is an amino acid derivative. Betaine can be produced endogenously from choline or ingested through exogenous dietary intake from beets, whole wheat, spinach, seafoods, and vertebrate organs [1]. The various biological properties of betaine include its participation in essential biochemical reactions, promoting growth, increasing muscle mass, lowering meat fat content, preventing osteoporosis, and protecting organs such as the liver, kidneys, and heart [2]. Betaine also exhibits therapeutic properties against many chronic diseases and tumors, indicating its human health benefits [1, 3]. Betaine has been approved by the European Commission for use in food and was initially approved by the U.S. Food and Drug Administration for treating homocystinuria, indicating its roles as a nutraceutical product and therapeutic agent [4]. Increasing evidence has indicated that betaine may have a protective effect on neuropsychiatric function and be a potential candidate for treating related disorders [5]. Despite growing awareness of the disability and mortality associated with neurological disease and psychiatric disorders, their prevention and treatment are still somewhat limited and unsatisfactory. Novel medicinal agents are being explored, and the beneficial roles of druggable nutraceuticals in preventing and/or treating these diseases have been emphasized [6]. Hence, this review aimed to provide updates on betaine as a promising medicinal nutraceutical with health benefits for neurological and psychiatric diseases (Fig. **1**).

## 2 METHODS

The literature search was conducted using the Web of Science, PubMed, and Embase databases. Search keywords included “betaine”, “neurological disease”, “psychiatric disease”, “epilepsy”, “Alzheimer's disease”, “vascular neurocognitive disorder”, “cognitive disorder”, “Parkinson's disease”, “stroke”, “multiple sclerosis”, “traumatic brain injury”, “depression”, “anxiety”, “schizophrenia”, “autism spectrum disorder”, “sleep disorder”, “fetal alcohol syndrome”, “syringomyelia”, “diagnosis”, “prevention”, and “side effect”. The search for keywords was set from the date of database establishment to the present. Firstly, all studies in English were searched one by one. Duplicate studies, retracted studies, and literature that were retracted and published again were excluded. Preclinical and clinical literature that met the requirements in this field were included. Subsequently, the abstracts and full texts of the articles that met the theme's requirements were carefully reviewed and studied. Finally, after a rigorous and careful selection and analysis, an objective, detailed description and discussion on this topic were conducted.

## 3 MECHANISMS OF BETAINE

### 3.1 Osmoregulation

Betaine is a highly soluble osmolyte that can reduce the ability of water molecules to dissolve proteins, thus stabilizing the natural protein structure and function. It can accumulate in cells without affecting their function, increase the cytoplasmic volume and free water content, prevent cell contraction under hypertonic conditions, and protect cells and proteins from osmotic stress, which is critical for maintaining internal homeostasis and protecting organs and tissues [1]. This unique osmoregulatory function of betaine is also conducive to maintaining the integrity of neuronal membranes, stability of neurons, and the osmotic pressure balance of brain tissue [7]. Betaine can prevent the misfolding and aggregation of proteins and maintain the soluble state of proteins through osmoregulation, which is beneficial for neuroprotection and the treatment of neurological diseases such as Alzheimer's disease. Betaine supplementation has been proposed as a prophylaxis against concussion in young athletes [8, 9].

### 3.2 Functioning as a Methyl Donor Regulating Metabolism, and an Antioxidant and Anti-inflammatory Agent

As a primary methyl donor, betaine is involved in several physiological processes. Betaine is one of the methyl donors (including other donors such as folates) in the Betaine-Homocysteine Methyltransferase (BHMT) pathway, which methylates Homocysteine (Hcy) to methionine and produces S-adenosylmethionine (SAM). With the decrease in Hcy, SAM is demethylated to S-adenosylhomocysteine (SAH). The ratio of SAM to SAH (SAM/SAH) influences Hcy methylation and Sulfur-containing Amino Acids (SAAs), which are involved in many metabolic pathways, including Glutathione (GSH) and protein synthesis, nutrient metabolism, and DNA methylation, possibly participating in the pathogenesis and treatment of some neuropsychiatric diseases (*e.g*., epilepsy, multiple sclerosis (MS), *etc*.) [1, 2, 10, 11].

Betaine and its related metabolic agents exhibit direct or indirect regulation through antioxidant or anti-inflammatory properties. Betaine methylates Hcy, thus reducing its plasma levels. Decreased blood Hcy levels are associated with a decreased risk of ischemic heart disease and stroke [12]. Betaine exerts antioxidant effects by participating in the methionine cycle and SAM production, thereby affecting SAA levels. These SAA levels, in turn, act as antioxidants by regulating GSH metabolism and reducing oxidative stress, while also enhancing non-enzymatic antioxidant defenses through the methionine-homocysteine cycle [13-16]. Betaine exerts anti-inflammatory effects by inhibiting mitogen-activated protein kinases, nuclear factor-inducible kinase/IκB kinase, and cysteine dioxygenase activities, maintaining thiol levels to inhibit the activity of nuclear transcription factor-κB (NF-κB) and various downstream sites, regulating the insulin pathway directly, alleviating chronic inflammation by restoring energy metabolism, and lowering immunoglobulin E levels [14, 17, 18].

### 3.3 Regulating Gamma-Aminobutyric Acid by Acting as a Substrate for the Transporter Protein

The Solute Carrier (SLC) family 6 is involved in betaine transport *via* SLC6A12, also known as Betaine/Gamma-Aminobutyric Acid (GABA) Transporter 1 (BGT-1), which is primarily expressed in the central nervous system [5, 19]. BGT-1 also transports the major inhibitory neurotransmitter, GABA, and is essential for maintaining its function [5, 19, 20]. Betaine has a higher affinity for BGT-1 than GABA and inhibits GABA uptake by binding to BGT-1 and interacting with GABA receptors [20]. Betaine can ameliorate stress-induced memory deficits in mice, which are inhibited by BGT-1/GABA transporter protein (GAT) 2 or GABAB receptor antagonism, suggesting that betaine’s potentially beneficial effects may be mediated by regulating the GABA system [21]. A recent study indicates betaine’s modulation of GABA homeostasis through the SLC6A1 transporter GAT1, thereby conferring neuroprotection against excitotoxicity [22]. Additionally, betaine suppresses the expression of GABA transaminase (for GABA degradation), the inhibition of which elevates GABA levels in human astrocytes. Thus, betaine regulates GABA levels and functions in various ways, thereby potentially benefiting the treatment of disorders such as epilepsy and Alzheimer’s Disease (AD) [23-25]. The mechanisms of betaine are summarized in Fig. (**2**).

## 4 BETAINE AND NEUROLOGICAL AND PSYCHIATRIC DISEASES

### 4.1 Epilepsy

Globally, approximately 65 million people suffer from epilepsy, and the adverse effects of current antiseizure drugs remain a challenge [26]. Betaine exerts anticonvulsant effects on seizures of different causes and can directly exert effects in the brain with intraventricular administration [27]. The enhancement of GABA inhibitory neurotransmitter transmission has been confirmed as one of the major targets of antiepileptic drugs [28, 29]. The direct antiepileptic effects of betaine may involve the BGT-1/GAT2 transporter, which is associated with GABAergic neurotransmission imbalance that leads to epilepsy [30]. With a higher affinity for BGT-1 than that for GABA, betaine decreases the transport and increases the accumulation of synaptic gap GABA, exerting an effect similar to the anticonvulsant effect of BGT-1 inhibitors, which possibly suggests a mechanism of betaine in treating epilepsy [24, 30, 31]. Betaine regulates the transmission of GABAergic inhibitory neurotransmitters through its unique function of competing with GABA for binding transporters, which provides a mechanistic target for the antiepileptic effect of betaine. Although seizure threshold and susceptibility are not significantly affected in BGT-1 knockout mice [32], both pharmacological inhibition of GAT1/BGT-1 and BGT-1-selective inhibition demonstrate anticonvulsant effects [30, 33, 34], and the antiepileptic effect of BGT-1 inhibitors in mice has been corroborated by a later study [24]. The seemingly inconsistent results from the above studies may be due to the interaction of other subtypes of GATs and the heterogeneity of different animal models, which require further investigation. Nevertheless, BGT-1 may still be a possible target involved in the mechanism for betaine’s antiepileptic effects. Betaine is a known methyl donor involved in various methylation reactions and important physiological functions. With a methyl-rich perinatal diet including betaine, the incidence of hereditary-absence epilepsy and comorbid depression reduces in the adult offspring of WAG/ Rij rats by about 50% as compared with that of 100% in the control rats, with the upregulation of genes associated with spike-wave discharges genesis and mesolimbic dopaminergic tone [35, 36]. WAG/Rij rats are commonly used as a genetic model of absence epilepsy. The onset of epilepsy in WAG/Rij rats is associated with increased spike discharge in the somatosensory cortex, which is associated with decreased expression of Hyperpolarization-activated Cyclic-Nucleotide-gated subtype 1 (HCN1), DNA Methyltransferase 1 (DNMT1), and Tyrosine Hydroxylase (TH) genes. A methyl diet rich in betaine increases their expression by enhancing the DNA methylation modification of these genes, thereby inhibiting spike discharges and ameliorating absence seizures [36]. In addition to the preclinical data, evidence for the clinical use of betaine in epilepsy stems from studies involving patients with methylenetetrahydrofolate reductase (MTHFR) deficiency, and when combined with some other nutrients, betaine ameliorates epilepsy symptoms or prevents its occurrence by reducing Hcy levels and restoring disturbed Hcy metabolism [37-39]. In a retrospective study of patients with MTHFR deficiency, nearly all patients received a combination of drugs, including betaine, and clinical symptoms associated with epilepsy improved after treatment. Therefore, the researchers speculate that betaine may be the most effective treatment [40]. In addition, treatment with combined betaine lessened the drug-resistant epileptic symptoms of a patient with 5-methyltetrahydrofolate-homocysteine S-methyltransferase gene deficiency continuously and effectively [41].

Thus, betaine combination regimens may have potential in the treatment of epilepsy for these patients. However, whether betaine alone is directly effective requires further studies to investigate additional underlying mechanisms, along with the clinical assessment of the betaine regimen to achieve optimal efficacy.

### 4.2 Neurocognitive Disorders

#### 4.2.1 Alzheimer's Disease

Alzheimer's Disease (AD) is a common neurodegenerative disorder and the main cause of dementia [42]. Potential therapeutic agents are being explored to improve the overall outcomes of patients with AD.

Increased plasma betaine might serve as a marker of higher instrumental activities of daily living score in elderly persons [43]. Betaine can have both direct and indirect effects on the triggering factors of AD pathogenesis. Betaine supplementation induces autophagy, thereby inhibiting the accumulation of amyloid-β (Aβ) [44], but promotes tau aggregation through osmoregulation directly [45]. However, betaine significantly reduces plasma Hcy level and reverses tau protein phosphorylation, and these effects outweigh its direct effects on tau aggregation, finally inhibiting Aβ accumulation and relieving AD [46, 47]. A systematic review also indicates that betaine supplementation causes decreases in Hcy levels, brain Aβ plaques, and p-Tau, and improvements in cognitive function, prognosis, and quality of life in patients with AD [45, 46]. A double-blind, placebo-controlled trial suggested that higher betaine concentrations are associated with improvement in cognitive function, indicating that betaine may improve cognitive impairment in AD patients [48]. The safety and tolerability of betaine in patients with AD have been investigated in an open-label, 24-week pilot study, lacking a control group. Thus, its efficacy is still unclear. However, these findings provided a basis for larger controlled trials with betaine in AD [49]. A study of the relationship between serum metabolites and the score of the Simple Mental State Examination Scale (MMSE) in dementia patients with AD found that betaine was positively correlated with the MMSE score [50]. A Mendelian randomized study of metabolites associated with AD and Parkinson's Disease (PD) found that tryptophan betaine may have a protective effect on both AD and PD [51]. However, a recent Mendelian randomized study has suggested that a decrease in betaine levels may be associated with a reduced risk of AD [52].

Preclinical studies have corroborated clinical studies and revealed additional targets involved in the underlying mechanisms. Betaine reduced Aβ levels by altering amyloid precursor protein processing, indicating its protective role in Aβ production [47]. Supplementation of betaine, combined with L-methylfolate and choline, reduces phospho-tau, Fyn, and demethylated protein phosphatase 2A levels, and mitigates learning and motor deficits in a mouse model of tauopathy, a feature of prevalent dementia, including AD [53]. In addition to regulating Aβ and tau, betaine inhibits Aβ-induced neuroinflammation in the microglia by inhibiting activation of the NOD-Like Receptor Pyrin domain containing-3 (NLRP3) inflammasome and NF-κB to reduce the expression of inflammatory factors (interleukin-1β, IL-1β; interleukin-18, IL-18; tumor necrosis factor-α, TNF-α) pathway [54], and attenuates Hcy-induced cognitive impairment by suppressing microglia pyroptosis *via* inhibiting the NLRP3/caspase-1/ gasdermin D pathway [55]. Betaine also prevents Aβ-induced impairment of memory possibly by suppressing oxidative stress in the hippocampus of a rat model through reducing the concentration or activity of hippocampal lipid peroxidation markers (Glutathione Peroxidase (GPx), Catalase (CAT), and Malondialdehyde (MDA)), as well as clearing Reactive Oxygen Species (ROS), and enhancing antioxidant activity by increasing levels of GSH [56]. Moreover, in addition to antioxidant activity, betaine also prevents the development of cognitive deficits in 3xTg mice, a genetic AD model, by improving synaptic structure and function as well [57]. Betaine also enhances synaptic plasticity by upregulating synaptic proteins, thereby attenuating Hcy-induced Alzheimer-like pathological changes and memory deficits [58]. Betaine also demonstrates preventive effects on cognitive deficits in Aβ-injected mice and reduces Sirtuin 1 (SIRT1) expression, which is a NAD+-dependent deacetylase that mediates metabolic reactions and contributes to the treatment of diseases such as aging [59].

Furthermore, BGT-1 may play a role in AD treatment with betaine. Evidence supports the transcriptional upregulation of the BGT-1 in the middle temporal gyrus of patients with AD, suggesting BGT-1’s involvement in AD pathogenesis [60]. A selective BGT-1 inhibitor reduces the preventive effect of betaine on Aβ-induced cognitive deficits, indicating that BGT-1 and betaine may protect against AD [23]. Additionally, excessive activation of Acetylcholinesterase (AChE) is associated explicitly with AD, and AChE Inhibitors (AChEIs) are employed as promising treatments [61]. However, activation of the cholinergic system by AChEIs leads to many adverse effects. Betaine in combination with glycine exhibits a significant binding affinity for AChE, inhibiting it by up to 99%, which preserves acetylcholine and alleviates AD while avoiding AChEIs’ side effects [62].

Thus, betaine shows promising potential for the prevention and treatment of AD, although further clinical trials with larger sample sizes are required before its broader applications can be made.

#### 4.2.2 Vascular Neurocognitive Disorders and Other Cognitive Disorders

Vascular Neurocognitive Disorders (VND) are a group of neurocognitive disorders caused by cerebrovascular injuries, and severe VND is termed as Vascular Dementia (VD). Therapies are mainly based on preventing and treating risk factors to reduce the incidence. In a rat model of VD with chronic cerebral hypoperfusion, betaine protects synaptic function by restoring the expression of Postsynaptic Density-93 (PSD-93), Postsynaptic Density-95 (PSD-95), and Microtubule-Associated Protein 2 (MAP2). It alleviates oxidative stress by inhibiting MDA and ROS and increasing Superoxide Dismutase (SOD) and GSH to rescue memory deficits [63]. In a clinical study of patients with acute ischemic cerebrovascular accidents, plasma betaine was inversely associated with cognitive impairment, further indicating its potential benefits [64]. In addition, in rodent models, betaine can further inhibit redox stress and prevent blood-brain barrier dysfunction by inhibiting Hcy-induced Matrix Metalloproteinase-9 (MMP-9) activity in the frontal cortex, thereby improving Hcy-induced memory impairment [65]. Betaine can also inhibit the over-activation of mouse hippocampal microglia, prevent microglia from becoming M1 type polarization, promote microglia to become M2 type polarization, and further reduce the mRNA expression of pro-inflammatory factors Interleukin-1 alpha (IL-1α), Interleukin-1 beta (IL-1β), chemokine C-C motif chemokine ligand 3 (CCL3), and Tumor Necrosis Factor-alpha (TNF-α), thus improving the cognitive impairment induced by chronic social isolation [66]. Betaine inhibits hippocampal interleukin-6 (IL-6), IL-1β, and TNF-α pro-inflammatory factors in hippocampus and serum, reduces MDA concentration and increase SOD activity to exert anti-inflammatory and antioxidant effects, and indirectly promotes the expression of hippocampal Phosphatidylinositol 3-kinase (PI3K) and protein kinase B (Akt), increases PI3K/Akt signal and reduces phosphorylated- Mechanistic Target of Rapamycin (p-mTOR) expression to exert neuroprotective effects, thereby improving streptozotocin-induced memory impairment [67]. Betaine reduces cognitive impairment in offspring rats by alleviating DNA damage induced by streptozotocin in the placenta of maternal-fetal rats [68]. Betaine downregulates the expression levels of IL-1β and TNF-α inflammatory factors in rat cerebral as well as cerebellar cortex and hippocampus, reduces MDA level, and elevates GSH, AChE, Nuclear Factor erythroid 2-like protein 2 (Nrf-2), and SOD gene expressions, thereby stimulating the cell reoxidation-reduction system to improve copper-induced memory impairment [69].

### 4.3 Parkinson's Disease

A fast-growing health-care issue with enormous global socioeconomic burden, Parkinson’s Disease (PD), is a common neurodegenerative disease [70]. Although both animal and clinical evidence considering betaine’s usage in PD are relatively lacking, there are some cues from previous studies. In the rotenone-induced PC12 cell model of PD, betaine inhibits cell apoptosis, increases cell viability and cell survival rate from 50% to 71% through alleviating mitochondrial dysfunction, inhibiting caspase-3/7 activation and production of reactive oxygen species, mitochondrial membrane depolarization and ATP depletion, and reducing nuclear fragmentation [71]. In PD rats, betaine elevates dopamine levels, glutathione peroxidase activity, and glutathione content in the cerebellum, and protects against hyperhomocysteinemia induced by levodopa and benserazide, indicating betaine’s potential benefits for PD [72]. Another study on the antioxidant effect of betaine on PD treatment drugs (levodopa and phenylselazid) also found that betaine can reduce the increase of plasma Hcy level caused by treatment drugs in rats, increase the activities of GPx, superoxide dismutase, and catalase in the brain of rats, and increase the level of glutathione. The results indicated that betaine was beneficial to antioxidant activity [73]. Single-nucleotide polymorphism analysis and data from genome-wide association studies have suggested a causal effect of betaine on PD progression evaluated by the Hoehn-Yahr stage, acting as a Trimethylamine N-Oxide (TMAO) precursor [74]. A Mendelian randomized study found that tryptophan betaine levels may be a protective factor for PD [51]. However, another Mendelian randomized study did not find a significant causal association between betaine and PD [75]. The same human study also found that the level of betaine detected in cerebrospinal fluid samples 4 hours after death in PD patients was one-third lower than that in the normal group [76]. Another metabolomic clinical study on the plasma and stool of PD patients found that the level of alanine betaine in feces of PD patients was lower than that of the normal group, which may be related to the direct metabolism of intestinal flora, indicating that intestinal ecological regulation may be associated with the pathogenesis of PD [77]. Nevertheless, no serious adverse effects have been found with betaine, and it is relatively safe with regular doses. Therefore, the results from this study need to be interpreted with caution and verified by further investigations. In patients with dementia and PD, homocystinuria and elevated Hcy levels in the frontal cortex are observed following levodopa administration, accompanied by a sharp decline in betaine levels. These changes correlate best with cognitive scores on the Mini-Mental State Examination, suggesting that betaine supplementation may reverse dementia in PD [78]. Although more direct evidence is required, betaine may potentially benefit both PD and its therapies.

### 4.4 Stroke

Stroke is a common neurological disorder with vascular impairment and is the second leading cause of death and the third leading cause of disability worldwide [79]. Derived from the choline metabolic pathway, which is important in the nervous system and atherosclerosis development, betaine may be beneficial for stroke.

The serum levels of betaine are decreased in patients with stroke [80]. In patients with ischemic stroke, each additional standard deviation of plasma betaine reduces the risk of subsequent stroke recurrence by 29%, suggesting the cerebrovascular protective significance of betaine [81]. A clinical study on the analysis of feces of patients for metabolites suggests that the metabolic pathways related to betaine biosynthesis were associated with recurrent stroke [82]. Patients with higher plasma betaine levels have a lower risk of cognitive impairment after ischemic stroke, supporting the promising prognostic role of betaine [64]. In patients with hypertension, a U-shaped correlation exists between baseline serum betaine and risk of the first ischemic stroke, indicating the importance of maintaining optimum betaine levels in these patients [83]. Moyamoya Disease (MDD), as a cerebrovascular malformation, is a risk factor for stroke. In a prospective study of 302 adult patients with MDD, as compared with their matched healthy individuals, the betaine serum levels were significantly decreased [84]. The risk of MDD is reduced by 0.952 of the odds ratio per 1 μmol increase in serum betaine, suggesting that high betaine levels may benefit in decreasing the risk of MDD [85]. Betaine treatment reduces cerebral infarct volume and neuronal apoptosis in a rat cerebral ischemia/reperfusion injury model induced by Middle Cerebral Artery Occlusion (MCAO), increases the expression of antioxidant enzymes such as Glutathione Peroxidase 4 (Gpx4) and Superoxide Dismutase 1 (SOD1) in frontal cortex, decreases the level of MDA in brain and serum, and elevates the level of SOD in brain and serum. Through antioxidant and anti-inflammatory mechanisms, betaine reduces H_2_O_2_-induced cell death in the PC12 cell line [86]. Betaine delays the onset of thrombus occlusion in mice and reverses adenosine diphosphate-induced platelet aggregation in whole blood samples, indicating its protection against coagulation events both *in vivo* and *in vitro* [87].

However, in a study of 412 identified patients with stroke and their matched controls, higher plasma levels of TMAO were found to be associated with a higher risk of stroke. However, as one of the precursors of TMAO, plasma betaine levels were not found to be significantly associated with the overall risk of stroke [88]. Moreover, analysis of the white matter hyperintensity volume in patients with ischemic stroke has revealed no association between betaine and cerebral small vessel disease [89]. Nevertheless, a study in young adults negatively associates betaine with cardiovascular disease among participants with lower estimated glomerular filtration rates, indicating the renal impact on the vascular effects of betaine [90]. In short, betaine may play a role in stroke prevention and risk control. Whether the inconsistencies in these studies were due to other physical condition factors, differences in study populations, the existence of a certain cutoff value for betaine plasma concentrations, or the lack of a linear correlation will be determined in further studies.

### 4.5 Multiple Sclerosis

Multiple Sclerosis (MS) is an autoimmune disease and is the most common cause of neurological disability in young adults [91]. MS involves dysregulated endogenous metabolism, wherein diets and nutrition play important roles [92]. As a methyl donor *in vivo*, betaine may help prevent MS. Cortical betaine concentrations are reduced in patients with MS and are associated with reduced Histone H3 trimethylation (H3K4me3) levels in neurons, possibly leading to defects in mitochondria and affecting neuronal energetics [93]. In MS mouse models, betaine supplementation increases methylation potential (SAM/SAH ratio) and H3K4me3 levels, enhances neuronal respiration, prevents axon damage, and alleviates neurological deficits, thereby providing neuroprotection and restoring epigenetic control [94], and attenuates MS pathogenesis by inhibiting production of dendritic cell-derived IL-6 and Th17 cell differentiation, and reduces leukocyte infiltration and central nervous system demyelination, suggesting betaine’s potential as a novel drug candidate for treating MS [95]. In a study of Cuprizone-induced MS model rats, the addition of betaine increased the levels of glutathione, glutathione peroxidase, and other antioxidant molecules in the cerebellum of rats, decreased the expression of endoplasmic reticulum stress-related proteins such as the Protein kinase RNA-like Endoplasmic Reticulum Kinase (PERK), Activating Transcription Factor 6 (ATF6), also reversed degeneration and demyelinating of cerebellar nerve cells and improved behavior and movement disorders in rats [96].

However, the evidence for betaine’s application in MS is lacking, and more direct evidence, especially from clinical studies, is required.

### 4.6 Traumatic Brain Injury

Traumatic Brain Injury (TBI) is the most prevalent neurological disorder and is predicted to be one of the top three causes of injury-related death and disabilities by 2030 [97, 98]. TBI can cause microglia activation, destruction of blood-brain barrier integrity, release of inflammatory factors, neuroinflammation, and other pathological changes [99, 100]. Betaine has physiological functions such as osmoregulation, maintaining the stability of cell structure, anti-inflammatory and anti-oxidative stress, which may be beneficial in combating the harm of post-traumatic brain injury. It has been found that mouse hippocampal slices promote the accumulation of betaine in an osmotic pressure-dependent manner, affecting the accumulation of other osmotic substances and altering osmotic pressure under isotonic and hypertonic osmotic stresses [7]. Animal studies have also found that betaine can inhibit the expression of pro-inflammatory factors (interleukin, tumor necrosis factor, etc.) at the gene level, transcription, replication, and protein level in the brain, inhibit the production of Reactive Oxygen Species (ROS), inhibit the activation of pro-inflammatory microglia, and enhance the activation of anti-inflammatory microglia. It can also enhance the activities of non-enzymatic antioxidant substances (GSH) and enzymatic antioxidant substances (SOD), playing an anti-inflammatory antioxidant effect and thereby reducing neuroinflammation, promoting the recovery of synaptic function, and protecting nerve function as well [63, 67]. Being neuroprotective, betaine may potentially improve the long-term outcomes of TBI. Betaine levels significantly decrease in the prefrontal cortex 24 hours after blast exposure in a TBI rat model under blast neurotrauma, suggesting a correlation between betaine and TBI development [101]. Proline betaine in cerebrospinal fluid may be a differential metabolite for TBI Disorder Of Consciousness (DOC) and non-TBI DOC [102]. In addition to its osmoregulatory and anti-inflammatory effects, betaine may also affect microvascular and body temperature regulation, which improves the pathological changes in TBI [103, 104]. Moreover, the intake of betaine before TBI injury reduces the levels of TBI biomarkers in the brain tissue, including caspase-3, cytochrome C, and neuron-specific enolase, suggesting betaine’s possible neuroprotective role in TBI [105]. A clinical study showed that plasma levels of betaine and methionine decreased in patients with severe TBI, indicating that TBI affected related circulation [106].

Betaine supplementation has been proposed as a prophylaxis against concussion in young athletes, particularly in female athletes [107]. More preclinical and clinical studies are needed to evaluate the long-term efficacy and safety of betaine in TBI.

### 4.7 Depression

Depression brings heavy global burdens, but approximately one-third of patients fail to respond to conventional antidepressants, and refractory depression presents great challenges in exploring superior treatment options [108, 109]. Betaine exerts antidepressant-like effects and enhances the antidepressant effects of other drugs, providing new cues. From the results of studies on metabolomic alterations in animal models of depression, cerebral betaine level decreases in the depressed animal [110], and circulating betaine level increases after antidepressant medication [111]. In preclinical studies, betaine significantly exhibits antidepressant-like effects in rodents and their models of depression, including ovariectomized mouse [112, 113], maternal separation rats [114], LPS-injected mice [115], mice subjected to chronic social defeat stress or chronic unpredictable mild stress [116, 117], mice exposed to zinc oxide nanoparticles [118], and complete Freund's adjuvant-induced pain-related depressed mice [119], and reduces the incidence of comorbid depression with genetic absence epilepsy in the adult offspring of WAG/Rij rats [36, 120]. Mechanisms of the antidepressant effects of betaine include mediation through the nitrergic and serotoninergic systems [113], elevating 5-hydroxytryptamine levels in the hypothalamus and hippocampus [112], inhibiting NLRP3 inflammasome activation and promoting the transition of microglia from the M1 to the M2 phenotype [115], altering the polarization of microglia and astrocytes to reduce neuroinflammation [119], exerting anti-inflammatory effects and ameliorating the abnormal diversity and composition of the microbiota in the host gut [116], and regulating the antioxidant capacity, Hcy levels, and brain-derived neurotrophic factor levels in the cerebral cortex and hippocampus [118]. Moreover, studies in mice indicate that betaine potentiates the antidepressant-like effects of ketamine but blocks its psychomimetic side effects [121]. Both co-treatment and post-treatment with betaine can effectively prevent and reverse the adverse behavioral manifestations and abnormalities in hippocampal synaptic plasticity after repeated ketamine use, with such interactions possibly being exhibited by regulating the N-methyl-D-aspartate receptor (NMDA) receptors [122].

Clinically, plasma betaine is associated with lower odds of prenatal depression and suicidal ideation in pregnant women [123]. Patients with Post-Stroke Depression (PSD) exhibit lower plasma betaine levels than those without PSD, and elevated plasma betaine levels are associated with a lower risk of developing PSD [124]. Betaine and SAM exhibit better results than amitriptyline in terms of both effectiveness and safety for mild depression after administration for 6 or 12 months [125]. Dietary betaine intake reduces the incidence of depression by 35% in overweight and obese patients [126]. Although there is a relatively abundant body of evidence to support the potential use of betaine for depression, more systematically controlled clinical trials with larger samples and high quality are required to confirm the direct use of betaine.

### 4.8 Anxiety

Anxiety disorders are the most common group of mental disorders, accounting for 3.3% of the global disease burden [127]. The onset of anxiety is potentially associated with the dysregulation of metabolism and nutrients. In patients with anxiety, the multiplication of plasma betaine, TMAO, and choline is a highly sensitive (85%) and specific (70%) diagnostic method for the disorder [128]. Mice with ulcerative colitis are accompanied by anxiety and depression-like symptoms. Betaine supplementation alleviates anxiety and depression symptoms in mice by inhibiting cGAS-STING signaling pathway (a key signaling pathway for neurogenesis), reducing DNA damage in the hippocampus, and mitochondrial dysfunction [129]. Betaine supplementation plays an antioxidant and anti-inflammatory role, protects brain function, and alleviates anxiety-like symptoms in rats by activating the nuclear factor erythroid 2-related factor 2 (Nrf2) pathway [130]. Betaine is one of the particularly increased urinary excretions associated with neurobehavioral disorders in children with refractory bedwetting and attention deficit hyperactivity disorder or anxiety, possibly modulating the renal and/or the central biophonic clock system as a renal osmotic agent and a methyl donor [131]. A study of 3299 adults has revealed reduced incidence of psychiatric disorders with high intake of methyl donors such as betaine, particularly in women and overweight or obese individuals, who are 67% less likely to experience anxiety [127]. However, a study in peripartum maternal mood has shown that plasma betaine concentrations during pregnancy are not associated with increased prenatal depression and anxiety [132]. Further studies are needed to elucidate the mechanisms and to determine if the inconsistencies in these findings are caused by differences in methodologies and target populations. Overall, the evidence for betaine use in anxiety is indirect and weak and needs to be better investigated.

### 4.9 Schizophrenia

Schizophrenia is a group of psychiatric disorders affecting approximately 1% of the global population and is among the top ten global causes of disability [133], and betaine may be a potential treatment alternative. Decreased betaine levels and betaine’s alleviation of elevated carbonyl stress in the postmortem brains of patients with schizophrenia suggest its involvement in the pathogenesis of schizophrenia and a causal link between betaine and oxidative stress conditions [134]. Decreased plasma betaine levels have been detected in patients with first-episode schizophrenia [135], and global DNA hypomethylation associated with decreased betaine levels in peripheral blood is common to these patients [136]. A recent systematic review of the search for plasma biomarkers related to schizophrenia has also suggested that plasma betaine levels are decreased in first-episode schizophrenia patients, indicating the benefit of betaine for schizophrenia [137]. Moreover, betaine supplementation significantly improves the positive symptoms, increases plasma methionine levels, and decreases plasma Hcy levels in patients with schizophrenia, indicating that betaine contributes to treatment, possibly by regulating methionine and Hcy levels [138]. Increased presence of betaine is associated with lower measures of insulin resistance in patients with schizophrenia [139].

Preclinical studies have demonstrated the benefits of betaine in treating schizophrenia as well. In Kif3b^+/-^ mice, a model of schizophrenia, betaine significantly alleviates schizophrenic traits related to neuronal morphogenesis and behavior, through decreasing collapsin response mediator protein 2 carbonylation, which enhances F-actin bundling and thus functionally compensates for KIF3 deficiency [140]. In animal models of schizophrenia established with Methamphetamine (MAP) or Phencyclidine (PCP), betaine supplementation suppresses MAP-induced behavioral sensitization, prevents PCP-induced behavioral abnormalities, and rectifies the antioxidant and pro-inflammatory responses induced by either MAP or PCP [134]. In addition, betaine blocks psychotomimetic behavior in ketamine-treated mice, suggesting its applicability in treating schizophrenia [121, 122]. Although the cellular and molecular mechanisms are unclear, further studies may validate the efficacy of betaine for treating schizophrenia or improving outcomes.

### 4.10 Autism Spectrum Disorders

Autism Spectrum Disorder (ASD) is a neurodevelopmental disorder that generally presents in early childhood [141]. Studies in children with ASD have indicated that plasma metabolic imbalance is consistent with impaired capacity for methylation and increased oxidative stress, and reduced concentrations of betaine have been observed in the urine [142, 143]. Genome-wide transcriptome analysis of offspring ASD risk markers in 300 pregnant mothers who had previously delivered children with ASD revealed that maternal plasma betaine and dimethylglycine concentrations were associated with a block of co-expressed genes relevant to children's later neurodevelopmental outcomes [144]. A study of serum metabolites has found increased serum betaine concentrations in patients with ASD compared to healthy controls [145]. Maternal obesity during pregnancy is associated with an elevated risk of ASD in the offspring, and the pregnant maternal plasma concentration of betaine correlates with significantly reduced preference for novelty and a change in emotional response to stressful situations in a nonhuman primate model [146]. In a preclinical mouse ASD model induced by prenatal valproic acid, significantly elevated serum Hcy levels, reduced serum betaine-Hcy methyltransferase expression, social impairment, and repetitive behavior can be reversed or attenuated by betaine [147]. However, another study on the ASD rat model induced by prenatal valproic acid exposure has found higher betaine levels in the prefrontal cortex than those in control rats, suggesting possible differences in central and peripheral regulatory mechanisms [148]. MTHFR is associated with an increased risk of ASD, and the offspring of MTHFR mice with symptoms of ASD have lower concentrations of betaine in the cerebral cortex than those without symptoms [149]. The results from a Mendelian randomization study have indicated no definite causality between betaine (as an intestinal metabolite) and ASD [150]. Although results from the above report are negative, they do not refute the role of other forms or sites of betaine and its benefits. Further controlled clinical trials are needed to evaluate the role of betaine in relieving symptoms and improving the long-term quality of life in patients with ASD.

### 4.11 Sleep Disorders

Sleep disorders are a common manifestation and/or cause of various neuropsychiatric disorders, with complex interactions with other psychosomatic diseases. Sleep disorders are associated with a variety of diseases, such as epilepsy, Parkinson's disease, depression, *etc.* [151-153]. Betaine is beneficial for the treatment of various neuropsychiatric disorders, suggesting that it may also be useful for sleep disorders. The mechanism of this effect includes restoring neurotransmitter transmission, reducing inflammatory responses, and regulating nutrients [154-156]. Betaine, as a dietary nutrient, may also have the potential to be applied in nutritional regulation for patients with sleep disorders, thereby improving sleep quality [157, 158]. A study on brain metabolic profiles in mice has revealed that a decrease in betaine level is associated with high sleep pressure, suggesting the potential of betaine as a novel biomarker of sleep pressure at the whole-brain level [159]. The metabolomics analysis of patients with Peritoneal Dialysis Restless Legs Syndrome (PD-RLS) and comorbid sleep disorders suggested N, N, N-trimethyl-L-alanyl-L-proline betaine as a potential biomarker for PD-RLS with good diagnostic and predictive ability [160]. The involvement of the cholinergic system in physiological sleep processes and the robust inhibitory effect of betaine on AChE suggest a role for betaine in regulating sleep. However, further confirmatory evidence is required [61, 62]. Overall, the evidence for betaine related to sleep disorders is not sufficient and needs more studies.

### 4.12 Fetal Alcohol Syndrome

Fetal Alcohol Syndrome (FAS) is a developmental disorder of the fetus caused by chronic, long-term maternal alcohol intake during pregnancy [161]. Betaine is beneficial to the normal maintenance of embryonic cell volume and the stability of histone and DNA methylation epigenetics due to its osmoregulation, methyl supply, anti-inflammatory, anti-oxidation, and a variety of neuroregulatory functions, which are beneficial to the normal development of embryos and the reduction of the risk of FAS [162-164]. In two studies on rat models, perinatal maternal ethanol exposure triggered brain cell death in the offspring, and betaine reduced Caspase-3, calpain levels, neuroapoptosis, and neurodegeneration, thus preventing FAS [165, 166]. Adding a maternal methyl donor-enriched diet including betaine before conception and throughout gestation effectively reduces the incidence and severity of alcohol-induced morphological defects in mouse offspring as well [167]. In an avian embryo model of FAS, betaine increases the rates of late-stage survival and decreases the head and body defects, suggesting the potential of betaine for attenuating prenatal alcohol-induced birth defects [168]. These animal studies indicated that betaine may prevent and treat FAS, but the clinical evidence is still limited.

### 4.13 Syringomyelia

Syringomyelia (SM) is a severe spinal cord disorder caused by congenital and acquired factors. In a hormonal injury-induced rat model of SM, there are significant increases in transporter BGT-1 and betaine levels, suggesting a possible association with SM pathogenesis [169]. In a rat model of post-traumatic SM, the BGT-1 is predominantly expressed in astrocytes in the vicinity of syrinxes, which uptake betaine through BGT-1 to regulate cell size. The osmo-protective effects of betaine are reversed by BGT-1 inhibitors, implicating the active role of betaine and BGT-1 in SM progression [170]. The enzyme Choline Dehydrogenase (CHDH), which is involved in the synthesis of betaine, was also upregulated during syrinx formation/expansion in SM injured spinal cords. It has been found in a post-traumatic SM rat model and *in vitro* cellular models that CHDH activity in the spinal cord provides locally synthesized betaine for osmoregulation in SM as well [171]. A further study in a rat post-traumatic SM model has confirmed the upregulation of betaine, BGT-1, and CHDH at the syrinx site, and alterations in osmolality at the syrinx site can be obtained by upregulation of betaine, implying extensive local osmoregulation activities at the site and possible future interventions with betaine in SM [172]. Further clinical evaluation is required to investigate the effects and mechanisms of betaine in SM before more definitive evidence is available.

### 4.14 Other Related Diseases and Functions

Betaine may be considered for neonatal brain health. Plasma betaine at the first 24 hours of life is significantly associated with cognitive and motor capabilities in a two-year neurodevelopmental evaluation for infants with neonatal encephalopathy, potentially serving as a predictor for neurological outcomes and adjunctive therapy in neonatal brain injury [173]. Betaine was found to be one of the suitable molecular biomarkers for Bayley Scales of Infant Development-III outcome prediction at 24 months in infants with neonatal hypoxic-ischemic encephalopathy [174].

Betaine attenuates chronic neuropathic pain in rats by inhibiting KIF17 (a kinesin protein)-NR2B-mediated neuroinflammatory signaling [175], and reduces pain response dose-dependently in mice, exerts anti-nociceptive and antioxidant activities, and interacts with opioidergic and GABA receptors [176].

Betaine can also improve motor function. Betaine prevents haloperidol-induced orofacial dyskinesia in rats, an analog of human Tardive Dyskinesia (TD), through antioxidant prevention of mitochondrial dysfunction and reducing neuroinflammatory and apoptotic markers in the striatum, suggesting its potential role as a novel therapeutic candidate for delaying or treating TD [177]. Betaine supplementation exerts ergogenic effects on strength and power performance in healthy males and improves the performance of athletes, including anaerobic and aerobic metabolism, muscle endurance, and body composition [107, 178, 179]. Betaine can delay age-related muscle loss by increasing SAM levels, thus promoting protein synthesis in male mice [180].

Moreover, betaine and fluorination modification significantly augment the Blood-Brain Barrier (BBB) crossing ability of cylindrical polymer brushes, providing a novel and safe strategy for enhancing the BBB-crossing ability of other drugs for the treatment of central nervous system diseases [181].

## 5 BETAINE FOR THE DIAGNOSIS AND PREVENTION OF NEUROLOGICAL AND PSYCHIATRIC DISEASES

### 5.1 Diagnosis

Although some metabolomics studies have identified altered betaine levels in many neurological and psychiatric disorders, betaine has not been used for clinical diagnosis for any disease so far. There have been some explorations in the usage of plasma betaine levels in the diagnostic models of AD, serum betaine to diagnose antenatal depression, urinary betaine levels for the diagnosis of bipolar depression, plasma N, N, N-trimethyl-L-alanyl-L-proline betaine as markers for the diagnosis of PD-RLS, and so on [160, 182-184]. A prospective study on the relationship between cognitive impairment and choline circulating metabolites in a total of 617 patients with ischemic stroke has shown that plasma betaine levels are negatively correlated with cognitive impairment [64]. A urinary metabonomic analysis of hypertensive patients finds that urinary proline-betaine levels are negatively correlated with systolic and diastolic blood pressure [185]. A clinical study assessing plasma metabolites in patients with incident cardiovascular disease reveals an inverse association between the baseline betaine-to-choline ratio and cardiovascular disease [186]. Nevertheless, further research is required to validate these diagnosis models, and the potential diagnostic feasibility of betaine for neuropsychiatric disorders seems premature due to insufficient specificity, sensitivity, and current technical difficulties.

A genetic analysis of betaine may aid in gene amplification and has been used to support or modify the genetic diagnosis of hereditary neurological diseases and syndromes, including Prader-Willi syndrome and Angelman syndrome, Fragile X Syndrome (FXS), and Huntington's disease [187-189]. Although the available evidence may not be sufficient to support the use of betaine in the diagnosis of neuropsychiatric disorders, the existing data can enlighten the research directions in the future.

### 5.2 Prevention

There has been no clinical evidence of betaine’s prevention of neurological and psychiatric diseases. Still, some preclinical studies in animal models have indicated its prophylactic effects against cytotoxic brain damage induced by chronic ethanol consumption [190], SAH-induced neurodegenerative impairments [191], amyloid β peptide or chronic-stress-induced cognitive deficits [23, 59, 192], LPS-induced memory impairment [193], stress-related anhedonia-like behaviors [116], PCP-induced schizophrenic behavioral abnormalities [134], neurotoxic effects of copper oxide nanoparticles [69], and the increase in expression of NMDA receptors in the brain induced by a high-fat diet [194]. Additionally, perinatal betaine pre-administration is beneficial for preventing congenital and developmental anomalies in the offspring, including mortality related to MTHFR deficiency [195], genetic absence epilepsy and comorbid depression [120], and craniofacial defects in animals [196], and exhibiting a lowest risk or prevention of Neural Tube Defects (NTDs) in human offspring [197, 198]. Moreover, early betaine administration prevents death and further diseases, normalizes psychomotor development, and improves the outcome in patients with severe MTHFR deficiency [199, 200].

A clinical study has found that the serum betaine level of patients with moyamoya disease reduces significantly [84]. A prospective clinical study has found that serum betaine levels decrease in patients with post-stroke depression [124]. A 1-year longitudinal clinical study involving more than 5000 patients explored the effects of dietary intake changes, such as betaine, on cardiometabolic and other functions. The study found that high levels of betaine intake can prevent obesity, regulate blood lipids, and improve cardiometabolic function [201]. Another clinical study has found that betaine supplements for football players over 14 weeks can help prevent the increase of inflammatory factors and white blood cells, thereby benefiting the functional exercise of the muscles [202]. In addition, prophylactic administration of betaine may have the potential to prevent concussion in young athletes [107]. These data may support the preventive effects of betaine, although there is still a lack of direct clinical evidence, which needs further studies.

## 6 SAFETY CONSIDERATIONS IN BETAINE APPLICATION

While betaine is generally considered a non-toxic dietary supplement, safety considerations remain important. The European Food Safety Authority (EFSA) recommends a maximum daily intake of 400 mg for adults and 6 mg/kg body weight for individuals under 18 [203, 204]. At therapeutic doses, betaine typically causes no severe adverse effects. However, it may induce reversible changes in blood biomarkers (*e.g*., decreased blood cells, plasma proteins, transaminases, serum calcium/phosphorus, and increased platelet count, total lipoprotein) and organ tissues (*e.g*., increased liver/kidney weight, hepatic vacuolation) [204-207]. Paradoxically, some studies demonstrate its hepatoprotective effects and improved lipid profiles [201, 208-210].

Common transient side effects include gastrointestinal discomfort (nausea, diarrhea) [49, 208, 211]. In homocystinuria treatment with anhydrous betaine (Cystadane^®^), rare cases of diarrhea, visual disturbances, and hypermethioninemia were reported, often attributed to non-compliance [212]. Other isolated reports describe halitosis, headaches, and one case of interstitial lung disease [213]. Notably, betaine-mediated homocysteine reduction increases methionine levels, potentially risking cerebral edema [214, 215], necessitating strict dosage adherence and methionine monitoring. A clinical trial noted elevated blood pressure with betaine-containing supplements, though confounding by other components (creatine, dendrobium) cannot be excluded [216]. Miscellaneous reports include fever, myalgia, and taste alterations [49, 204, 208, 217], though topical betaine may alleviate xerostomia [218, 219].

While betaine itself is not allergenic, its production using *Saccharomyces cerevisiae* poses a theoretical sensitization risk for yeast-allergic individuals [204]. Current evidence shows no reproductive toxicity, carcinogenicity, or significant adverse outcomes in clinical/preclinical studies [204, 220-222]. Therefore, when used within recommended guidelines, betaine demonstrates a favorable safety profile. The possible side effects of betaine are shown in Table **1**.

## 7 LIMITATIONS, CONTROVERSIES, AND PERSPECTIVES

Current research on betaine presents several critical limitations that hinder a comprehensive understanding of its biological roles and clinical potential. Firstly, existing studies exhibit some inconsistencies in metabolite analysis outcomes, potentially attributable to variations in participant demographics, experimental designs, methodologies, and protocol specifications. Secondly, the absence of standardized betaine administration regimens across investigations further complicates comparative analysis, while the frequent co-administration of betaine with other methyl donors or pharmacological agents obscures its specific contributions. Thirdly, most clinical data remain preliminary, characterized by small sample sizes and unsystematic evaluation frameworks, significantly limiting the reliability of conclusions. Additionally, it is difficult to directly translate the results of animal studies to human clinical data.

While current reports suggest intriguing medical potential for betaine, the fragmented nature of existing evidence, including inconsistent conclusions across studies and insufficient replication of results, creates substantial interpretative challenges. This preliminary stage of investigation, coupled with limited high-quality clinical data and a lack of consensus regarding its therapeutic mechanisms, underscores the necessity for rigorous, well-designed research initiatives. Systematic exploration through multicenter trials with standardized protocols, controlled experimental variables, and expanded sample sizes is essential to clarify betaine's biological functions, optimize therapeutic applications, and enable evidence-based clinical recommendations. Such efforts would not only resolve current discrepancies in the literature but also facilitate the development of standardized intervention strategies for this promising natural compound.

Before the full therapeutic potential of betaine is realized, future research should focus on the following aspects. Firstly, the most credible underlying mechanisms of betaine and its interaction with the central nervous system in neurological and psychiatric diseases need to be elucidated. Secondly, it is equally important to extrapolate preclinical findings into clinical studies for a better understanding of betaine’s role. Thirdly, further high-quality clinical studies on the applications of betaine should be well-designed and sufficiently powered, and large-scale Randomized Controlled Trials (RCTs) are warranted to establish robust clinical evidence. Fourthly, comparative investigations should be prioritized to evaluate betaine both as monotherapy and in combination regimens, aiming to develop standardized protocols for single medication and optimize synergistic treatment strategies. Moreover, additional RCTs focusing on betaine’s clinical applications in disease diagnosis and prevention are needed. Clinical studies should also be conducted to determine the optimal doses of betaine for treating various diseases, thereby enabling the development of a standardized regimen. Lastly, larger-scale trials on betaine in the diagnosis and prevention of diseases should be carried out to promote its clinical applications. Furthermore, more comprehensive safety assessments are also required.

## CONCLUSION

Growing evidence supports the benefits of betaine in neurological and psychiatric health, and its role in the etiology, development, diagnosis, prevention, and treatment of related diseases. In addition to its regular properties, betaine exerts effects with considerable potential breadth. The possible specific mechanism of betaine in the central nervous system includes the regulation of GABA associated with its transporters, and thus, downstream targets and neural pathways. There is relatively adequate evidence supporting the efficacy and potential applications of betaine for epilepsy, AD, and depression. Additional benefits of betaine in neurological and psychiatric health and diseases include safety, ample sources, ease of use, and affordability. Betaine demonstrates a generally favorable safety profile, with most adverse effects being mild and clinically manageable when administered within recommended dosage ranges. While current evidence remains limited, particularly from large-scale clinical trials, this review consolidates existing knowledge to provide insights for future research, contributing to the further development of betaine’s applications in neurological and psychiatric health and diseases.

## Figures and Tables

**Fig. (2):**
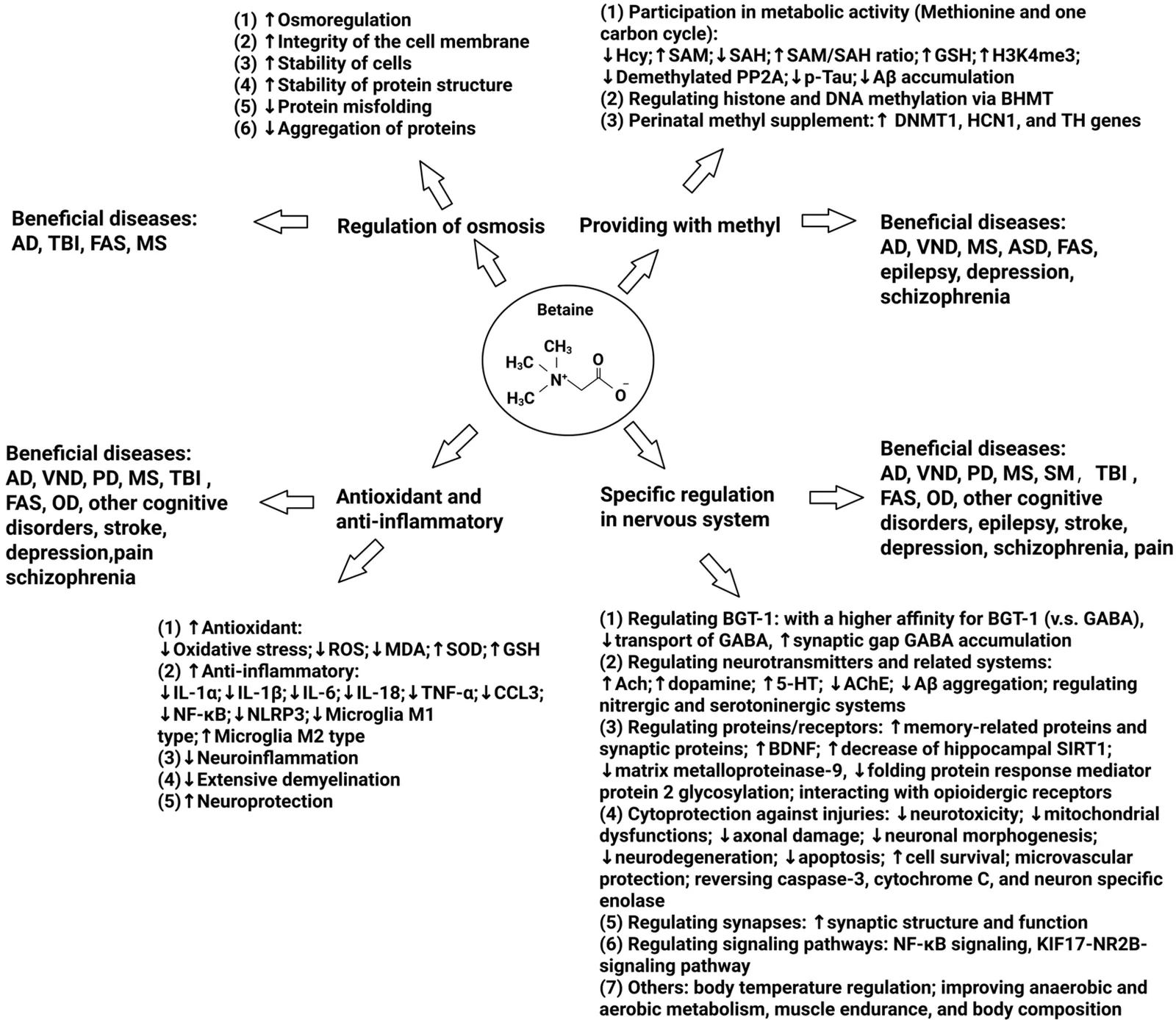
Summary of mechanisms of betaine for neurological and psychiatric diseases based on up-to-date data. **Abbreviations**: Aβ, amyloid-β; ACh, acetylcholine; AChE, acetylcholinesterase; AD, Alzheimer’s disease; ASD, autism spectrum disorder; BDNF, Brain-derived neurotrophic factor; BGT-1, betaine/GABA transporter 1; BHMT, betaine-homocysteine methyltransferase; CCL3, chemokine c-c motif chemokine ligand 3; DNMT1, DNA methyltransferase 1; FAS, fetal alcohol syndrome; GABA, gamma-aminobutyric acid; GSH, glutathione; HCN1, hyperpolarization-activated cyclic-nucleotide gated subtype 1; Hcy, homocysteine; H3K4me3, histone H3 trimethylation; 5-HT, 5-hydroxytryptamine; IL-1α, interleukin-1 alpha; IL-1β, interleukin-1 beta; IL-6, interleukin-6; IL-18, interleukin-18; KIF17, kinesin superfamily motor protein 17; MDA, malondialdehyde; MS, multiple sclerosis; NF-κB, nuclear transcription factor-κB; NLRP3, NOD-like receptor pyrin domain containing-3; NR2B, N-methyl-D-aspartate receptor subtype 2B; OD, orofacial dyskinesia; PD, Parkinson’s disease; PP2A, protein phosphatase 2A; ROS, reactive oxygen species; p-Tau, phosphorylated tau; SAH, s-adenosylhomocysteine; SAM, s-adenosylmethionine; SIRT1, Sirtuin 1; SM, syringomyelia; SOD, superoxide dismutase; TBI, traumatic brain injury; TH, tyrosine hydroxylase; TNF-α, tumor necrosis factor-alpha; VND, vascular neurocognitive disorders.

**Table 1:** Possible side effects of betaine.

**Blood System**	**Vital Organs**	**Gastrointestinal System**	**Others**
Blood cells↓	Liver weight↑	Nausea↑	Visual disturbances
Plasma proteins↓	Kidney weight↑	Diarrhea↑	Headaches
Transaminases↓	Hepatic vacuolation↑	-	Halitosis↑
Serum calcium↓	-	-	Lung disease
Serum phosphorus↓	-	-	Potentially risking
Platelet count↑	-	-	Cerebral edema
Total lipoprotein↑	-	-	Fever
Methionine↑	-	-	Myalgia
-	-	-	Taste alterations
-	-	-	Risk of elevated blood pressure

## LIST OF ABBREVIATIONS

ACh: Acetylcholine
AChE: Acetylcholinesterase
AChEIs: Acetylcholinesterase Inhibitors
AD: Alzheimer’s Disease
ASD: Autism Spectrum Disorder
Aβ: Amyloid-β
BBB: Blood-brain Barrier
BGT-1: Betaine/GABA Transporter 1
BHMT: Betaine-homocysteine Methyltransferase
CCH: Chronic Cerebral Hypoperfusion
CHDH: Choline Dehydrogenase
CSDS: Chronic Social Defeat Stress
DMG: Dimethylglycine
DNMT1: DNA Methyltransferase 1
FAS: Fetal Alcohol Syndrome
FXS: Fragile X Chromosome Syndrome
GABA: Gamma-aminobutyric Acid
GATs: GABA Transporter Proteins
GSH: Glutathione
H3K4me3: Histone H3 Trimethylation
HCN1: Hyperpolarization-activated Cyclic-nucleotide Gated Subtype 1
Hcy: Homocysteine
HP: Haloperidol
LPS: Lipopolysaccharide
MCAO: Middle Cerebral Artery Occlusion
MDA: Malondialdehyde
MS: Multiple Sclerosis
MS-PCR: Methylation-sensitive Polymerase Chain Reaction
MTHFR: Methylenetetrahydrofolate Reductase
NF-κB: Nuclear Transcription Factor-κB
NLRP3: NOD-like Receptor Pyrin Domain Containing-3
NTDs: Neural Tube Defects
OD: Orofacial Dyskinesia
PCP: Phencyclidine
PD: Parkinson’s Disease
PD-RLS: Peritoneal Dialysis-restless Legs Syndrome
PSD: Post Stroke Depression
SAA: Sulfur-containing Amino Acid
SAH: s-adenosylhomocysteine
SAM: s-adenosylmethionine
SLC: Solute Carrier
SM: Syringomyelia
TBI: Traumatic Brain Injury
TD: Tardive Dyskinesia
TH: Tyrosine Hydroxylase
TMAO: Trimethylamine N-oxide
VD: Vascular Dementia
VND: Vascular Neurocognitive Disorders
